# Data gaps for symptoms of myocardial infarction in transgender and gender diverse patients: a brief literature review and call to action

**DOI:** 10.3389/fpubh.2026.1856093

**Published:** 2026-06-05

**Authors:** Darren Schoenike, Caitlin B. Yoakum, Joanne L. Peterson

**Affiliations:** 1 Arkansas College of Osteopathic Medicine, Arkansas Colleges of Health Education, Fort Smith, AR, United States; 2 Department of the Anatomical Sciences, Arkansas Colleges of Health Education, Fort Smith, AR, United States

**Keywords:** cardiovascular disease, gender affirming hormone therapy, heart attack, heart attack symptoms, Myocardial Infarction, transgender and gender diverse

## Abstract

Cardiovascular disease is the leading cause of death in the United States and has garnered several avenues of efforts to reduce incidence and mortality in patient populations. There has also been an increase in reported populations of transgender and gender diverse individuals as well as a reported increased risk for cardiovascular disease in this population. Despite this, there is no current research assessing warning signs of myocardial infarction and no standard of care exists for educating transgender and gender diverse individuals regarding their heart health. This article reviews the known literature on interactions between gender affirming hormone therapy and cardiovascular disease and highlights the need for more research of myocardial infarction onset symptoms in transgender and gender diverse populations to provide better heart health education and care.

## 1 Introduction to transgender and gender diverse identities

The National Center for Transgender Equality describes transgender and gender diverse (TGD) individuals as those who do not identify with the gender associated with their sex assigned at birth. A transgender man is someone who was assigned female at birth and a transgender woman is someone who was assigned male at birth. A cisgender person is someone whose gender identity aligns with the gender associated with their sex assigned at birth (1). However, there is no singular correct way to identify and experience being TGD; it is an umbrella term that encompasses an infinite number of possibilities to experience gender. The authors would like to briefly recognize the existence of intersex persons and their overlapping experiences between both typical cisgender and TGD experiences. While the experiences of intersex identities will not be explored in this paper, that is not to undermine their importance within this discussion. Nonbinary and gender diverse identities are also relevant to this conversation, but due to an even greater paucity of research for these populations, we focus on the traditional gender binary in terms of identity and hormone ranges to draw more supported comparisons within the existing data regarding heart health and disease between cisgender and transgender populations, while still referring to the latter as the TGD population.

In speaking about the difference between sex and gender, original statements are made in accordance with defining sex to be the identity assigned at birth based on natally acquired physical traits, and gender to be the identity explicitly expressed by participants. Hormone levels are regarded within standard physiologic cisgender ranges when mentioned. When referencing data and findings cited from previous publications, the language used by those specific studies is mirrored, which may or may not be reflective of the actual difference between the sex and gender of the participants.

### 1.1 Transitioning

The common ways TGD individuals will ‘transition to a different gender' are to use a different name, different pronouns, or dress in a way that more closely aligns with how they wish to express their gender identity; these are all potential aspects of *social transition* (2). Comparatively, *medical transition* involves practices that require a licensed professional to access. This includes gender affirming hormone therapy (GAHT) where one takes hormones to achieve the physical features typically associated with their gender identity. Masculinization with testosterone can be injected (most common), implanted as a pellet, orally, or applied transdermally. Feminization is via estradiol which can be administered orally, transdermally, or injected. Anti-androgens such as spironolactone or cyproterone acetone are taken orally (2). The duration of GAHT is a personalized decision which can range from continuous lifelong treatment to only until certain desired irreversible effects are obtained. Multiple bodies of research have displayed the importance of access to GAHT for TGD individuals as a form of life saving treatment with its ability to increase quality of life and reduce poor mental health outcomes (3–5). Medical transition can also involve gender-affirming surgeries which include a wide variety of procedures from altering facial features, breast enhancement or reduction, and alteration or removal of sex organs (2). A TGD individual may choose to undergo as few or as many of the possible ways to transition as they determine they need.

## 2 Cardiovascular disease

Cardiovascular disease (CVD) is the leading cause of death in the United States (US), claiming nearly 700,000 lives per year and receiving over $3 billion in research funding each year (6, 7). From understanding risk factors and prevalence to developing more efficacious treatment methods and prevention, the goal is promoting health and longevity in as many people as possible. Receiving proper education on heart health equips patients with knowledge that allows them to play an active role in making healthy choices by means of preventative care (8).

### 2.1 Myocardial infarctions in cisgender populations

One type of CVD - myocardial infarction (MI; i.e., heart attack) - is described as range of possible symptoms from chest pain, discomfort, pressure or squeezing, sweating, pain in the neck or jaw, shoulder or arm pain, back pain, nausea, shortness of breath, and light-headedness (9, 10). Historically, cisgender women have experienced poorer diagnosis accuracy and higher mortality rates despite having lower incidence rates of MI compared to cisgender men (11–14). Research over the past few decades has shown conflicting results with its attempts to uncover if there are true differences in the symptoms experienced by cisgender men and cisgender women. It has been thought that cisgender women are more likely to exhibit the “atypical” symptoms which Greenslade (15) has defined as epigastric or back pain characterized as burning, stabbing, or similar to indigestion,vs. “typical” pain being located in the chest, arm, or jaw, and characterized as dull, heavy, tight, or crushing (11–13, 16, 17). Newer articles show that cisgender women may be experiencing “typical” chest pain more frequently than cisgender men, along with experiencing an overall increased number of total associated symptoms when presenting with an MI (16, 18). Others are critiquing the usefulness of “typical”vs. “atypical” designation for symptoms and suggest at minimum providing reference to the group with which these designations apply to, along with cautioning against the idea of there being a “correct” or “standard” way for a patient to present with an MI (17).

A lack in patient education on MI symptom identification may contribute to differences patients experienced in the clinical setting. An article using data from the 2001 Behavioral Risk Factor Surveillance System showed that of the 61,018 participants, chest pain was the most widely known symptom of a heart attack, with 95% of respondents correctly identifying that option. 89% identified pain/discomfort in the arm or shoulder and 87% for shortness of breath as heart attack symptoms (12). 65% of participants indicated that feeling weak, light-headed, or faint were symptoms. One symptom was found to be recognized at significantly different rates between cisgender men and women, with 46% of cisgender men vs. 55% of cisgender women, was knowing that pain in the jaw, neck, or back were possible symptoms of an MI (12). Survey data from over 225,000 Korean participants claimed significant associations between inadequate knowledge of CVD warning signs and participants with the following characteristics: “older age, male gender, lower education level, lack of regular exercise, unmarried status, unemployment, poor economic status, poor health behaviors…poor psychological status… and the presence of hypertension or dyslipidemia” (19).

None of the articles on CVD and MI mentioned thus far have included data from TGD individuals, yet many of those risk factors for poor CVD education are experienced at disproportionate rates in this population (20). The current recommendations from the Fourth Universal Definition of Myocardial Infarction from 2018 include the use sex-specific cardiac troponin levels to aid in the diagnosis of MI and this may prove to be especially helpful in improving the MI diagnosis and treatment inequity faced by cisgender women and potential differences seen in TGD patients (18, 21, 22). Coupled with the findings of inconsistent MI symptoms and diagnoses in cisgender women and men, this has prompted our inquiry as to if there are differences in the MI symptoms experienced in TGD populations along with the consideration as to if exogenous hormones play a role.

## 3 Methods

This article is a review that pulls from currently published literature and data on the rate of cardiovascular disease within the TDG population and highlights the lack of existing information regarding MI symptoms experienced by TGD populations. A search amongst multiple electronic databases such as Elsevier and PubMed was used to gather results. Relevant studies were searched for via keywords and phrases such as “transgender”, “transgender and gender diverse”, “heart attack”, “myocardial infarction”, “heart attack symptoms”, “myocardial infarction symptoms”, “onset symptoms”, “heart disease”, “high sensitivity cardiac troponins”, along with the Boolean operator “AND”. Important to note is that this search yielded no results when searching for data regarding MI symptoms amongst TGD populations.

Articles were included if they included discussions or background on CVD in TGD populations. Articles that both did or did not examine the relationship between GAHT and CVD were included. Peer-reviewed original research, reviews, case reports, and conference podium presentations were included. Exclusion criteria were animal studies, editorials, opinion pieces, and those that were not focused on the physical health of TGD populations. No informed consent or IRB approval was required due to only using previously collected data from public academic sources.

## 4 Effects of gender affirming hormone therapy

Recent data show that of the estimated 1.4 million TGD adults in the US, about 55% of them access GAHT as a part of their medical transition (23). With social prevalence and awareness of TGD individuals rising, there has been increased accessibility to resources for TGD patients to medically transition but still with limited research regarding long term use of GAHT in the TGD population (24). Outside of the desired masculinizing or feminizing effects from GAHT, there are other wide spread effects on the body, such as metabolic processes including lipid levels (2). Current research has noted some of these effects such as testosterone tending to cause a significant increase in low-density lipoprotein and triglyceride levels along with a decrease in high-density lipoprotein levels, whereas estrogen only showed an increase in triglyceride levels (25). Cisgender women tend to have more favorable lipid panels than cisgender men in the general population, so it is not surprising that TGD patients on GAHT would follow similar patterns (25). Newer data suggests that GAHT leads to a change in physiologic levels of high-sensitivity cardiac troponin levels (hs-cTn) in healthy transgender men and women, with transgender men's hs-cTn levels rising to similar levels as cisgender men and transgender women's decreasing to similar levels of as cisgender women (26, 27). However, it cannot yet be determined if there is a significant difference in hs-cTn levels between transgender men and women after GAHT has altered them from their original baseline (28). The effects of GAHT on lipid and hs-cTn levels have warranted much consideration for the relationship GAHT may play in heart health in the TGD population.

## 5 Cardiovascular disease in transgender and gender diverse individuals using gender affirming hormone therapy

While there is growing research looking at the rates of CVD such as MI in TGD populations, there is no current research that considers the potential differences in MI symptoms within TGD populations despite the concerning data that suggests this population experiences an increased prevalence CVD (29–34). Restar and Streed (35) note that in accordance with the American Heart Association, there are eight key health factors and behaviors that account for heart health: tobacco use, weight, diet, exercise, blood sugar levels, cholesterol levels, blood pressure, and sleep quality. Yet these factors are often overlooked in TGD individuals, and the focus becomes reduced to their gender-affirming care, with particular emphasis placed on exogenous GAHT (35). Veale (29) utilized a national survey of TGD participants with the New Zealand Health Survey to assess stress-related health conditions and indicators and found that TGD participants had a greater likelihood of having hypertension (63%), myocardial infarction (98%), stroke (104%), and hypercholesterolemia (148%) at some point in life. Veale (29) and Ong et al. (30) have also shown that in US-specific data, TGD men and women reported increased MI incidents compared to their cisgender counterparts (29, 30). However, other research has suggested contradictory findings such as that TGD men may have lower MI incidence than TGD women, or TGD individuals having the same or similar CVD risk factors when compared to the general population, and that different GAHT (i.e., testosterone vs. estrogens) might have closer associations with other types of CVD such as stroke or venous thromboembolism (2, 31, 32). There is also conflicting data as to whether it is the use of specific GAHT types and/or certain dosages that could be a potential cause of the increased rates of CVD (29).

Streed et al. (24) bring attention to the fact that most current studies looking at CVD rates focus on younger TGD adults who began GAHT as young adults rather than middle age or older adults. When considering the two main types of GAHT, testosterone and estradiol, there is less conclusive evidence showing testosterone GAHT is causing lipid panel changes in a way that contributes to CVD at increased rates than the average cisgender male experience. Specifically, Thompson (36) points out that there are no published studies comparing the prevalence of dyslipidemia between TGD men and cisgender men while also controlling for hormone use although some case reports do blame the use of GAHT in certain doses or time periods for causing MIs over time (36, 37).

When looking at those who use estrogen GAHT, research has more conclusively shown a measurable increase in CVD (33). Case studies have shown instances of recurrent deep vein thrombosis (DVT) in individuals as young adults with no prior family history (38). Other data have demonstrated a higher risk of venous thromboembolism in TGD women using either oral or injected estrogen (39), and a general increase in CVD risk with the injected ethinyl estradiol form of exogenous estrogen in both cisgender and TGD women (24, 25, 29, 32, 39). Data thus far tend to suggest TGD women have higher incidence of MI compared to cisgender women but at similar rates to cisgender men (24, 32). However, older TGD women on GAHT appear to have an especially high CVD risk, regardless of the age they began GAHT or the duration of use (24). This is particularly interesting when paralleled with the findings that hormone replacement was protective in reducing CVD in cisgender women with early menopause or post-hysterectomy, yet increased risk in older postmenopausal or post-hysterectomy cisgender women (24). It has also been suggested that a combination of both estrogen and progesterone hormone replacement increased incidence of MI and stroke in cisgender women as well (24). Overall, most data agree that estrogen GAHT is associated with increased CVD risk, but whether it is truly the result of the GAHT rather than a “legacy effect of natal sex” has not yet been established (32, 34).

These studies highlight Restar and Streeds' (35) critique that TGD individuals often have their heart health outcomes reduced to their use of GAHT rather than other known risk factors. A retrospective study looking at TGD women on estrogen GAHT found that nearly all patients who experienced VTE had additional risk factors for thromboembolism including smoking, immobilization, or a clotting disorder (40). It has also been noted that smoking appears to be a better risk factor predictor of MI rather than estrogen or testosterone GAHT use (29). Ong et al. (30) and Streed et al. (20) both use the Minority Stress and Intersectional Transgender Multilevel Minority Stress Models as frameworks to show the effects of stress on TGD patients' rates of CVD as psychosocial, behavioral, and clinical factors that all come into play in perpetuating both psychological and physiological stress. Also imperative to recognize is that there is a large volume of systemic discrimination within the healthcare setting and a general lack of understanding surrounding TGD identities that contributes to poorer quality healthcare for this community (41).

### 5.1 Cardiovascular risk prevention and protective factors in transgender and gender diverse individuals

Streed et al. (20) note connections between modifiable risk factors and the role that changing them may aid in cardiovascular risk prevention, such as physical activity levels being lower in TGD populations as a whole yet those using GAHT are more likely to be engaging in physical activity and that the “best predictor of participation is high body satisfaction”. Their use of the Intersectional Transgender Multilevel Minority Stress Model also shows how resiliency-promoting factors at “individual, interpersonal, and structural levels” such as pride in identity, self-esteem, social support, and identity affirmation can serve as protective factors to mitigate the negative effects of the stressors faced by TGD individuals. Ong et al. (30) recommend that primary preventative care for TGD utilize focused and affirming approaches to encourage risk modification lifestyle changes whereas secondary prevention may need to rely on health education based on the patient's individual health history and along with specialized endocrine and cardiology care due to a lack of current guidelines to provide direction for primary care clinicians. Primary prevention may also consider social determinants of health (SDOH) as chronic psychosocial and environmental stressors have been shown to affect physiologic pathways that increase chronic inflammation and have been linked to poorer cardiovascular morbidity in marginalized communities such as Black populations and Hispanic/Latinx populations (35). Multiple different stressor types within SDOH can intersect with TGD populations, further increasing their risk for chronic inflammation to influence their cardiovascular health (35). Specifically, the Minority Stress Model has been used to explain higher levels of cortisol in TGD populations, yet it has also been shown that those levels decreased after beginning GAHT (42).

Protective factors such as social support, community connectedness, familial acceptance, access to legal support for transition, and rejection of internal stressors via self-affirmation have been shown to decrease psychological stress which in turn reduces poor mental health outcomes as well as improve physiologic resilience (35). A previous study considered various risk and protective factors and their impact on multiple health outcomes in TGD populations and reported a statistically significant positive effect of social support on the general physical health in TGD participants based on self-reported responses to items from the SF-8 Health Survey (43). Feelings of community belongingness were also associated with being protective but were not statistically significant (43). However, there are no current studies that have assessed or quantified the impact of protective factors on cardiovascular health outcomes specifically. Nonetheless, the protective factors associated with psychologic and physiologic resilience suggest that affirmation across multiple domains is necessary to most effectively improve cardiovascular health (35).

## 6 Conclusion

This review aims to highlight the need for further investigation of MI onset symptoms in TGD populations so that we can effectively and accurately educate this community on their heart health. Regardless of cause, the current outcome is that the TGD community is facing disparity in terms of heart health. And despite this increased prevalence, there has yet to be any research that seeks to observe the warning signs of MI in this population. Beach and Streed (44) comment that a TGD woman who has medically transitioned, “with the exception of chromosomes, has shifted to fall into the typical female designation. These are not merely cultural changes; they have physical and biological relevance”. With the widespread effects that GAHT has on the body, it warrants the question: Does GAHT change the anticipated onset signs of a heart attack so that they will be more closely aligned with the sex associated with that dominant hormone? Current studies argue that due to insufficient evidence and lack of official clinical guidelines regarding this topic, there is no reason that TGD men and women should be screened for CVD any differently than their cisgender counterparts (25, 44). Cardiologists and clinicians should also be mindful to not presume that GAHT is the main factor contributing to a TGD patient's CVD risk (35, 45). When diagnosing an MI in a TGD individual, Wang et. al. (46) has shown that there has not been a difference in outcomes or safety when using the hs-cTn threshold that is in congruence with the individual's gender identity rather than sex assigned at birth. While these are helpful starting points for providing adequate care to the TGD community, it does not give direction on how to tailor the appropriate education that should be given to individuals using GAHT or to healthcare providers in regard to MI symptoms. Education plays a pivotal role in preventive medicine for heart health, and more research is needed to better equip TGD individuals with the knowledge and warning signs that can potentially save their lives (13). Increasing the opportunity to educate TGD patients about their cardiovascular health in clinical settings requires adequate training and cultural competency to facilitate a gender-inclusive clinical environment and decrease the negative healthcare experiences that contribute to poorer cardiovascular health behaviors and outcomes in TGD populations (20, 30).
